# Synthesis of cyclic *N*^1^-pentylinosine phosphate, a new structurally reduced cADPR analogue with calcium-mobilizing activity on PC12 cells

**DOI:** 10.3762/bjoc.11.289

**Published:** 2015-12-22

**Authors:** Ahmed Mahal, Stefano D’Errico, Nicola Borbone, Brunella Pinto, Agnese Secondo, Valeria Costantino, Valentina Tedeschi, Giorgia Oliviero, Vincenzo Piccialli, Gennaro Piccialli

**Affiliations:** 1 Dipartimento di Farmacia, Università degli Studi di Napoli Federico II, Via D. Montesano 49, 80131, Napoli, Italy; 2 Dipartimento di Neuroscienze e Scienze Riproduttive ed Odontostomatologiche, Università degli Studi di Napoli Federico II, Via Pansini 5, 80131 Napoli, Italy; 3 Dipartimento di Scienze Chimiche, Università degli Studi di Napoli Federico II, Napoli, Italy; 4 Institute of Protein Biochemistry, National Council Research of Italy, Via Pietro Castellino 111, 80131 Napoli, Italy

**Keywords:** calcium mobilization, cIDPR analogues, cyclic ADP-ribose (cADPR), cyclization

## Abstract

Cyclic *N*^1^-pentylinosine monophosphate (cpIMP), a novel simplified inosine derivative of cyclic ADP-ribose (cADPR) in which the *N*^1^-pentyl chain and the monophosphate group replace the northern ribose and the pyrophosphate moieties, respectively, was synthesized. The role played by the position of the phosphate group in the key cyclization step, which consists in the formation of a phosphodiester bond, was thoroughly investigated. We have also examined the influence of the phosphate bridge on the ability of cpIMP to mobilize Ca^2+^ in PC12 neuronal cells in comparison with the pyrophosphate bridge present in the cyclic *N*^1^-pentylinosine diphosphate analogue (cpIDP) previously synthesized in our laboratories. The preliminary biological tests indicated that cpIMP and cpIDP induce a rapid increase of intracellular Ca^2+^ concentration in PC12 neuronal cells.

## Introduction

**Figure 1:**
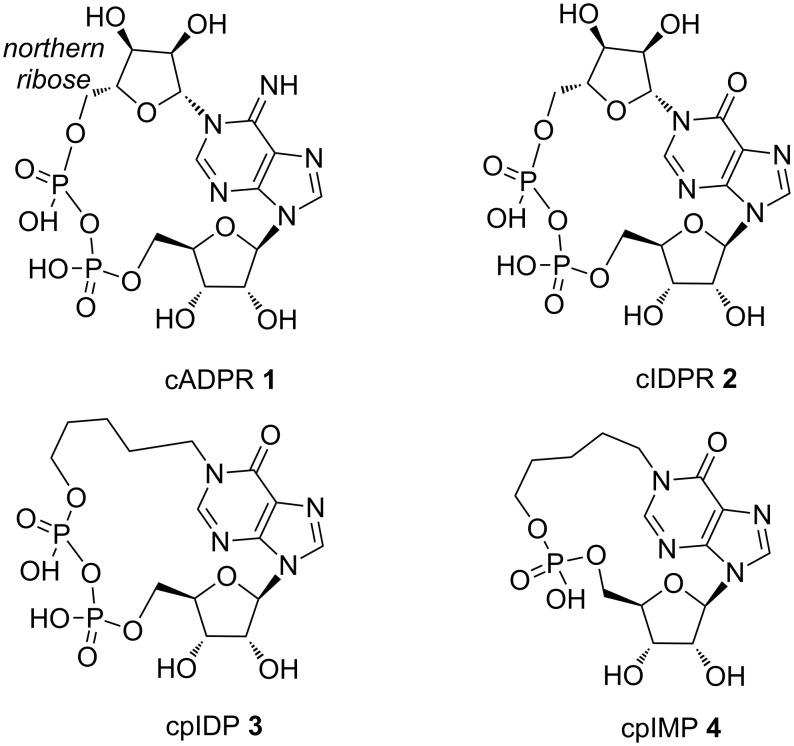
Structures of cADPR (**1**), cIDPR (**2**), cpIDP (**3**) and cpIMP (**4**).

Nucleosides and nucleotides (NNs) are widely used as key intermediates and important core structures in the field of synthetic medicinal chemistry [1–2]. They represent versatile synthetic building blocks towards the synthesis of biologically relevant compounds such as antiviral and antineoplastic drugs [3–8], antibiotics and antifungal agents [9–11]. Furthermore, several NNs act as potent second messengers involved in the regulation of key metabolic pathways [12]. Among these NNs there is the cyclic ADP-ribose (cADPR **1**, Figure 1), a metabolite strictly involved in the homeostasis of cellular calcium ions. cADPR is a second messenger that activates the ryanodine receptors of the sarcoplasmic reticulum and mobilizes Ca^2+^ ions in many cell types of protozoa, plants, animals and humans [13].

Furthermore, there is a strong evidence that cADPR is an important second messenger in the nervous system where it is involved in the handling of Ca^2+^ ions that control several functions. Indeed, the administration of cADPR to cell cultures produces three patterns of response in terms of variation of intracellular concentration of calcium ions ([Ca^2+^]_i_): (a) a rapid response after direct microinjection of the messenger into the cells; (b) a slow variation of [Ca^2+^]_i_ when cADPR is added to the cell culture medium; c) a progressive potentiation of [Ca^2+^]_i_ increasing due to a depolarization. In fact, cADPR induces Ca^2+^ release from presynaptic and postsynaptic intracellular stores and plays an important role in the activity-dependent synaptic plasticity, including long-term depression [14]. In addition, enzymes able to catalyze the hydrolysis of cyclic ADP-ribose to ADP-ribose are expressed ubiquitously in the mouse brain. Specifically, wild-type mice show the highest cyclase activity in the hypothalamus, and then in the cerebellum, cerebrum and posterior pituitary [15].

Unfortunately, the lability of the N-1 glycosidic bond of cADPR towards enzymatic and/or non-enzymatic hydrolysis to ADP-ribose, even in a neutral aqueous solution, greatly hinders the studies aimed at elucidating its physiological role [16]. Several enzymes involved in the metabolism of cADPR have been described. Among them is the ubiquitous ADP-ribosyl cyclase, an enzyme first isolated from *Aplysia Californica* [17]. Using the *Aplysia* ADP-ribosyl cyclase many metabolite analogues of cADPR have been produced starting from NAD^+^ and NADP^+^ [18–21]. However, the specificity of the enzymatic cyclization mechanism drastically limits its applicability for enzymatic or chemo-enzymatic procedures. For this reason, to obtain new cADPR derivatives the exploitation of chemical synthetic strategies is still necessary. A lot of modifications regarding the northern and southern ribose as well as the purine base of cADPR have been proposed so far [22–23]. Matsuda and co-workers were the first who synthesized new analogues of the cADPR in which the adenine base was replaced by a hypoxanthine ring [24]. This kind of modification produced the cyclic inosine diphosphate ribose (cIDPR) **2** which proved to be stable in hydrolytic physiological conditions and showed significant Ca^2+^ mobilizing activity, thus fostering the synthesis of a variety of cIDPR analogues. In particular, the N1, N9 and C8-substituted cIDPR were the most interesting [24–33]. In the last few years several cIDPR analogues were also synthesized in our laboratory [34–37]. Among these, the *N*^1^-pentyl analogue cpIDP (**3**, Figure 1) [34] showed interesting activity on the PC12 cell line previously differentiated with the Nerve Growth Factor (NGF) (data not previously published). Starting from these data, we here report the synthesis and the preliminary biological activity of the new cyclic *N*^1^-pentylinosine monophosphate (cpIMP) (**4**, Figure 1), in which the pyrophosphate group of **3** was replaced by a monophosphate moiety connecting the southern ribose with the *N*^1^-pentyl chain. The here reported results significantly contribute to the understanding of the role played by the pyrophosphate moiety of cADPR and cIDPR analogues on the mobilization of Ca^2+^ ions. To date, such structure-activity relationship has only been poorly investigated [38–41]. We anticipate here that compounds **3** and **4** induce the same effect on Ca^2+^ mobilization in NGF-differentiated PC12 cells. In particular, both compounds produced a transient increase of intracellular concentration of Ca^2+^ when added to the cells, thus demonstrating their ability to cross the plasma membrane.

## Results and Discussion

### Chemistry

**Figure 2:**
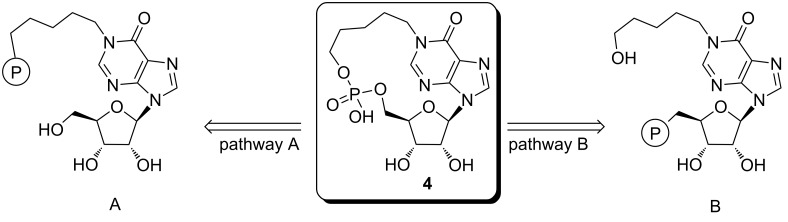
Synthetic strategies explored in the cyclization step via phosphodiester bond formation.

The key step for the preparation of all cADPR/cIDPR analogues is the macrocyclization via pyrophosphate bond formation, which is usually performed by joining the two phosphate moieties at the end of the multistep synthesis [24–29]. Similarly, the preparation of the new cpIMP derivative **4** could be performed by the cyclization of a monophosphate precursor via phosphodiester bond formation. For this purpose we investigated both the possible synthetic strategies (pathways A and B, Figure 2) in which the phosphate group (or its synthon) has been attached either on the end of the *N*^1^-pentyl chain (precursor A) or on the 5’-ribose position (precursor B).

**Scheme 1:**
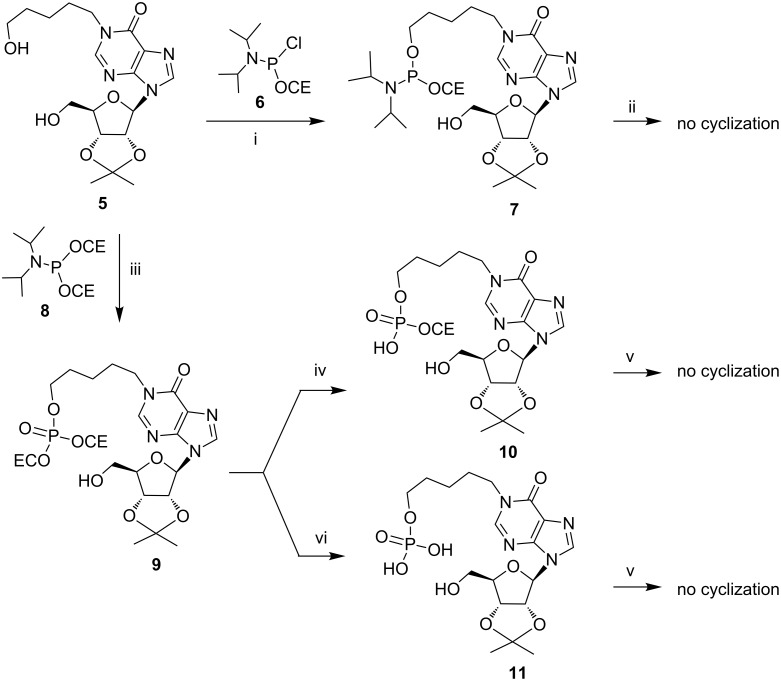
i) (iPr)_2_NP(OCE)Cl, DIPEA, THF, 1 h, rt; ii) 1) 1*H*-tetrazole, THF, 2) *t*-BuOOH, 2 h, rt; iii) 1) (iPr)_2_NP(OCE)_2_, 1*H*-tetrazole, THF, 2 h, rt, 2) *t*-BuOOH, 2 h, rt; iv) TEA/pyridine, 1:1 v/v, 16 h, rt; v) activating agent (EDC in DMF or DCC in DMF or MSNT in pyridine) 16 h, rt; vi) conc. aq NH_4_OH, MeOH, 50 °C, 16 h.

At first, to obtain the cpIMP (**4**) we followed the synthetic pathway A by using the strategy reported in Scheme 1 that employed the *N*^1^-ω-hydroxypentylinosine derivative **5** as the starting material [34]. Compound **5** was phosphorylated on the ω-hydroxyalkyl function by using the phosphitylating chloro-amidite agent **6**. The reaction of **5** with **6** furnished the sole regioisomer **7** equipped with the reactive phosphorous(III) group. Unfortunately, the activation of the phosphoramidite function with 1*H*-tetrazole aimed at inducing the cyclization on the 5’-OH ribose function produced only a complex mixture. No traces of the target cyclic compound were detected after the usual phosphorous oxidation step.

This failure prompted us to use the alternative phosphitylating reagent bis(cyanoethyl)phosphoramidite **8**, which, after the regioselective reaction with the 5’-hydroxyalkyl function of **5** led to the phosphotriester product **9** after the phosphorous oxidation with *t*-BuOOH. Starting from **9** we explored two synthetic routes, differing for the degree of esterification at the phosphate moiety, to achieve the cyclization of the 17-membered ring of **4**. The treatment of **9** with a mixture of triethylamine/pyridine furnished the phosphodiester product **10** in almost quantitative yield. Instead, the complete removal of both 2-cyanoethyl groups of **9** with concentrated aqueous ammonia gave the phosphomonoester **11**. Unfortunately, neither linear precursors **10** nor **11** underwent the expected cyclization step, even when treated with the most common phosphate activating agents (EDC, DCC, MSNT) in very diluted conditions. In our opinion, the target intramolecular cyclizations failed because of the poor mobility of the 5’-OH ribose function, as well as because of the unfavourable *anti* conformation of the N-glycosidic bond induced by the presence at the N1 position of the purine base of the bulky ω-phosphate adduct formed with the activating agent.

**Scheme 2:**
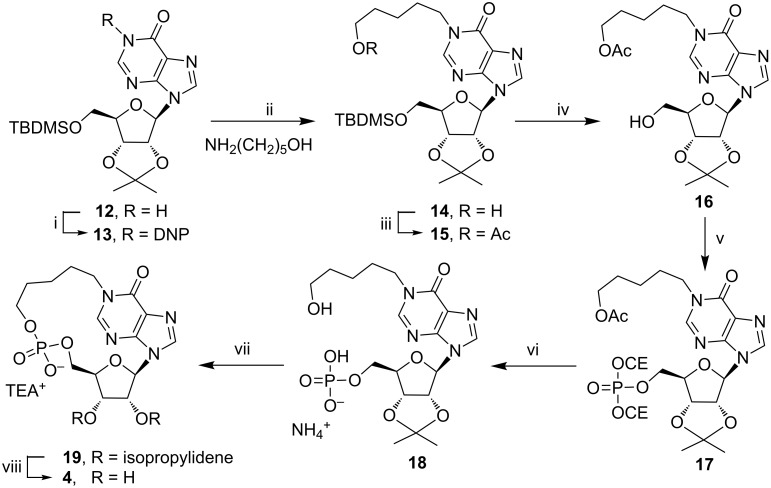
i) DNCB, K_2_CO_3_, DMF, 4 h, 80 °C; ii) 5-aminopentan-1-ol, DMF, 16 h, 50 °C; iii) Ac_2_O, pyridine, 2 h, rt, iv) NH_4_F, MeOH, 16 h, reflux; v) 1) (iPr)_2_NP(OCE)_2_, 1*H*-tetrazole, THF, 2 h, rt, 2) *t*-BuOOH, 2 h, rt; vi) conc. NH_4_OH_(aq)_, MeOH, 50 °C, 16 h; vii) EDC, DMF; viii) TFA, H_2_O, 16 h, rt.

For this reason, we decided to switch to the synthetic pathway B (Figure 2), in which the monophosphate group is installed at the 5’-ribose position. This strategy, reported in Scheme 2, used as the starting material the 5’-TBDMS-2’,3’-*O*-isopropylideneinosine (**12**). The protected inosine **12** was initially converted into the *N*^1^-dinitrophenyl derivative **13**, which, after reaction with the 5-aminopentan-1-ol, furnished the *N*^1^-ω-hydroxypentylinosine derivative **14** [35]. This compound was acetylated on the ω-hydroxy function (compound **15**) and then deprotected on the 5’-hydroxy function thus obtaining **16**. The phosphorylation of the 5’-OH function of **16**, by using the (iPr)_2_NP(OCE)_2_/*t*-BuOOH system, already used in the preparation of compound **9**, furnished the 5’-*O*-phosphotriester inosine derivative **17**. The treatment of **17** with concentrated aqueous ammonia allowed the removal of both the OCE phosphate protecting groups together with the acetate function, thus obtaining the key intermediate **18** as triethylammonium salt after HPLC purification. The derivate **18**, dissolved in DMF at the final concentration of 2 mM was treated with EDC (1.2 equiv) and the reaction allowed to stand at room temperature for 48 h. From this mixture it was possible to isolate cyclic compound **19** (30% cyclization yield) whose structure was confirmed by NMR and high-resolution mass analyses. Eventually, the treatment of compound **19** with aqueous 20% TFA afforded the target compound **4**.

### Ca^2+^-mobilizing activity of **3** and **4** in PC12 cells

**Figure 3:**
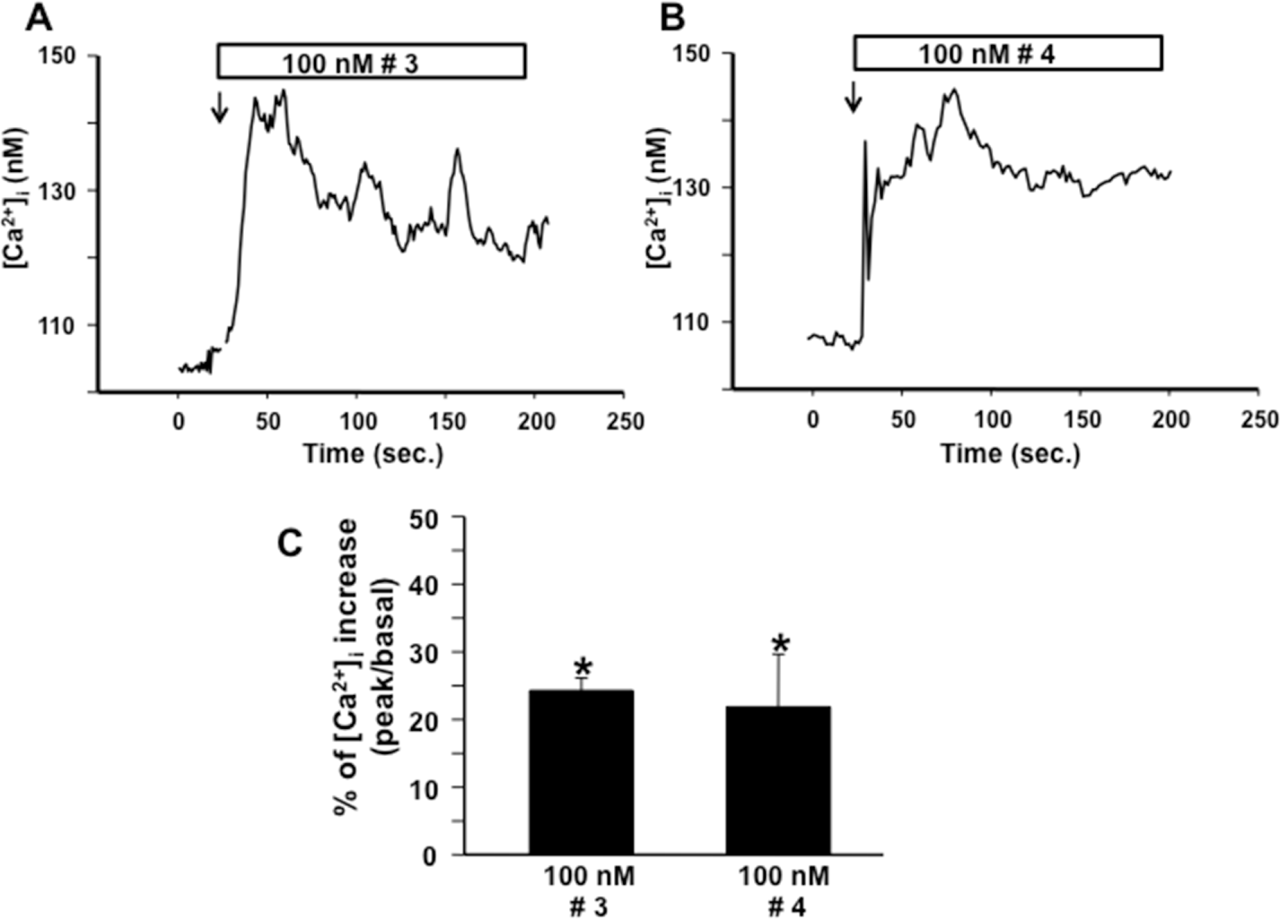
Effect of **3** and **4** on intracellular [Ca^2+^] in NGF-differentiated PC12 cells. (A) and (B): representative single-cell traces of the effect of **3** and **4** (100 nM) on [Ca^2+^]_i_,. (C): quantification of [Ca^2+^]_i_ increase calculated, after the addition of each compound, as the percentage change of plateau/basal value. Each bar represents the mean (±S.E.M.) of the values obtained in three independent experimental sessions. For each experiment, 10 to 40 individual cells were monitored. *, *P* < 0.05 versus basal level.

To study the biological activity of compounds **3** and **4**, we evaluated their effect on the mobilization of Ca^2+^ ions in PC12 cells differentiated with NGF. Interestingly, compounds **3** and **4** caused a rapid and transient increase of the intracellular [Ca^2+^] ([Ca^2+^]_i_) when added to the medium at the concentration of 100 nM (Figure 3). This pattern of response could be ascribed to the initial release of Ca^2+^ ions from the intracellular organelles followed by a depolarization-induced Ca^2+^ influx. The biological assays also confirmed that the cIDPR analogues **3** and **4** retained the ability to pass the plasma membrane of neuronal cells.

## Conclusion

cADPR is a second messenger synthesized by neuronal cells that modulates the Ca^2+^ homeostasis in the nervous system [42]. Unfortunately, cADPR is characterized by a low ability to cross the plasma membranes. This behaviour hinders the studies on the effects of cADPR on cell functions. To overcome this limitation and to understand the role played by the pyrophosphate bridge on the biological activities of cADPR, we synthesized the lipophilic derivative cpIMP (**4**) in which the pyrophosphate group of **3** was replaced by a monophosphate moiety connecting the southern ribose with the *N*^1^-pentyl chain. We compared the activity of **4** in modulating the concentration of [Ca^2+^]_i_ with that of the previously synthesized cpIDP (**3**). To study the effect of **3** and **4** on [Ca^2+^]_i_, these compounds were added to PC12 cells previously differentiated with NGF at the concentration of 100 nM. Both compounds caused a fast and transient increase in [Ca^2+^]_i_. This pattern of response could be ascribed to the initial release of Ca^2+^ from intracellular organelles followed by a depolarization-induced Ca^2+^ influx. The reported preliminary results indicate that **3** and **4** possess almost the same activity, thus indicating that the role of the pyrophosphate bridge is not stringent and that the introduction of an alkyl chain in the N1 position of the purine base improves the permeation of the cell membrane by passive diffusion or through an active uptake system expressed on the membrane.

## Experimental

### General

All solvents were dried by standard methods and all reactions were carried out under inert atmosphere (argon or nitrogen). All reagents were obtained and used from commercial sources (Sigma-Aldrich, Germany) without further purification. ^1^H and ^13^C NMR experiments were performed using a Varian Mercury Plus 400 MHz spectrometer in CD_3_OD, D_2_O, CDCl_3_ and acetone-*d*_6_ solvents. Chemical shifts are reported in parts per million (δ) relative to residual solvents signals: CD_2_HOD 3.31, HOD 4.80, (CD_3_)(CD_2_H)CO 2.09 for ^1^H NMR and CD_3_OD 49.0 for ^13^C NMR. ^31^P NMR experiments were carried out on a Varian Unity INOVA 500 MHz instrument in CD_3_OD solvent using 85% H_3_PO_4_ as an external standard (0 ppm). High performance liquid chromatography (HPLC) was performed using a Jasco UP-2075 Plus pump equipped with a Jasco UV-2075 Plus UV detector and a 4.8 × 150 mm C-18 reversed-phase column (particle size 5 µm) eluted with a linear gradient of CH_3_CN in 0.1 M triethylammonium bicarbonate (TEAB) buffer (from 0 to 50% in 45 min, flow 1.3 mL/min). UV spectra were recorded on a Jasco V-530 UV spectrophotometer. High-resolution MS spectra were recorded on a Bruker APEX II FT-ICR mass spectrometer using the electrospray ionization (ESI) technique. Column chromatography was carried out on silica gel-60 (Merck, 0.063–0.200 mm) or on C-18 reversed-phase silica gel-60 (Merck, 0.040–0.063 mm). Analytical TLC analyses were performed using F_254_ silica gel plates (0.2 mm thick, Merck). TLC spots were detected under UV light (254 nm).

### Cell cultures and [Ca^2+^]_i_ measurements

PC12 cells, grown on plastic dishes in RPMI medium composed of 10% horse serum, 5% FBS, 100 UI/mL penicillin and 100 μg/mL streptomycin, were differentiated in neurons with NGF (50 ng/mL; 7 days). Cells were cultured in an atmosphere of 5% CO_2_. The culture medium was changed every 2 days. For microfluorimetric studies with Fura 2-AM, cells were seeded on glass coverslips (Fisher, Springfield, NJ, USA) coated with poly-L-lysine (5 μg/mL) (Sigma, St. Louis, Missouri, USA) and used at least 12 h after seeding. Intracellular Ca^2+^ concentration ([Ca^2+^]_i_) was measured by single cell computer-assisted video QImaging [43]. Briefly, differentiated PC12 cells cultured on poly-L-lysine-coated glass coverslips were loaded with 10 µM Fura-2AM for 1 h at 22 °C in Krebs–Ringer saline solution containing the following: 5.5 mM KCl, 160 mM NaCl, 1.2 mM MgCl_2_, 1.5 mM CaCl_2_, 10 mM glucose, and 10 mM HEPES-NaOH, pH 7.4. At the end of the loading period, the coverslips were placed in a perfusion chamber (Medical System, Greenvale, NY, USA), mounted on a Zeiss Axiovert 200 microscope (Carl Zeiss, Germany) equipped with a FLUAR 40X oil objective lens. The experiments employed a digital imaging system composed of a MicroMax 512BFT cooled CCD camera (Princeton Instruments, Trenton, NJ, USA), LAMBDA 10-2 filter wheeler (Sutter Instruments, Novato, CA, USA), and Meta-Morph/MetaFluor Imaging System software (Universal Imaging, West Chester, PA, USA). After loading, the cells were illuminated alternately at 340 and 380 nm by a Xenon lamp. The emitted light was passed through a 512 nm barrier filter. Fura-2AM fluorescence intensity was measured every 3 s. Forty to sixty-five individual cells were selected and monitored simultaneously from each cover slip. Results are presented as the cytosolic Ca^2+^ concentration. Calibrations used the relation of Grynkiewicz et al. [44] assuming that the KD for Fura-2AM was 224 nM.

## Supporting Information

File 1 Structural characterizations.
