# Supplementary material for: Synthesis of cyclic N1-pentylinosine phosphate, a new structurally reduced cADPR analogue with calcium-mobilizing activity on PC12 cells
Source: Beilstein J Org Chem. 2015 Dec 22;11:2689–95. doi: 10.3762/bjoc.11.289 (PMC4734301; doi:10.3762/bjoc.11.289)
Supplement: File 1 — Structural characterizations. [file Beilstein_J_Org_Chem-11-2689-s001.pdf]

Supporting Information

for

Synthesis of cyclic N^1-pentylinosine phosphate, a new structurally reduced cADPR analogue with calcium-mobilizing activity on PC12 cells

Ahmed Mahal[‡,1], Stefano D'Errico[‡,1], Nicola Borbone[1], Brunella Pinto[1], Agnese Secondo[2], Valeria Costantino[1], Valentina Tedeschi[2], Giorgia Oliviero[*,1], Vincenzo Piccialli[3] and Gennaro Piccialli[1,4]

Address: [1]Dipartimento di Farmacia, Università degli Studi di Napoli Federico II, Via D. Montesano 49, 80131, Napoli, Italy, [2]Dipartimento di Neuroscienze e Scienze Riproduttive ed Odontostomatologiche, Università degli Studi di Napoli Federico II, Via Pansini 5, 80131 Napoli, Italy, [3]Dipartimento di Scienze Chimiche, Università degli Studi di Napoli Federico II, Napoli, Italy and [4]Institute of Protein Biochemistry, National Council Research of Italy, Via Pietro Castellino 111, 80131 Napoli, Italy

Email: Giorgia Oliviero - golivier@unina.it

*Corresponding author

[‡]These authors contributed equally

Structural characterizations

2’,3’-*O*-Isopropylidene-*N*[1]-[5’’-*O*-(2-cyanoethyl-*N,N*-diisopropylphosphoroamidite)pentyl] inosine (7)

Compound **5** (70 mg, 0.18 mmol) was coevaporated with anhydrous benzene (3 × 1 mL) and then dissolved in anhydrous THF (1.0 mL). To this solution DIPEA (0.040 mL, 0.21 mmol) and then $(iPr)_2NP(OCE)Cl$ (0.050 mL, 0.21 mmol) were added and the mixture was stirred at room temperature for 1 h. After the completion of the reaction (TLC monitoring: hexane/AcOEt, 6:4), the mixture was diluted with AcOEt (10 mL) and extracted with buffer phosphate solution at pH 7 (2 × 10 mL). The organic layer was separated and dried over anhydrous Na_2SO_4. The solvent was evaporated under reduced pressure and the crude was purified over 2% KOH coated silica gel column eluted with increasing amounts of AcOEt in hexane (from 20 to 40%). The fractions were collected and the solvents removed under reduced pressure, affording pure compound **7** (75 mg, 70%) as a 1:1 mixture of diastereomers.

Oil; ^{1}H NMR (400 MHz, acetone-d_6, assignments by HH-COSY experiment) δ 8.34 (s, 1H, 2 x 2-H), 8.23 (s, 0.5H, 8-H), 8.20 (s, 0.5H, 8-H), 6.22-6.18 (two overlapped doublets, *J* = 3.7, 2.8 Hz, 1H, 2 x 1’-H), 5.40-5.33 (two overlapped multiplets, 1H, 2 x 2’-H), 5.13-5.06 (two overlapped multiplets, 1H, 2 x 3’-H), 4.46-4.39 (two overlapped multiplets, 1H, 2 x 4’-H), 4.11 (two overlapped triplets, *J* = 7.1 Hz, 2H, 2 x CH_2N), 4.00-3.80 (complex signal, 4H, 2 x CH_2OP and 2 x $CNCH_2CH_2OP$), 3.70-3.60 (complex signal, 2H, 2 x 5’-$H_{a,b}$), 2.84-2.78 (two overlapped multiplets, 2H, 2 x CHN), 2.75 (two overlapped triplets, *J* = 6.1 Hz, 2H, 2 x CH_2CN), 1.88-1.77 (two overlapped multiplets, 2H, 2 x CH_2), 1.73-1.62 (two overlapped multiplets, 2H, 2 x CH_2), 1.57 (two overlapped singlets, 3H, 2 x CH_3), 1.54-1.44 (two overlapped multiplets, 2H, 2 x CH_2), 1.36 (two overlapped singlets, 3H, 2 x CH_3), 1.18 (d, *J* = 3.4 Hz, 6H, 4 x CH_3CHN), 1.16 (d, *J* = 2.2 Hz, 6H, 4 x CH_3CHN). ^{31}P NMR (202 MHz, CD_3OD) δ 149.5 (s). HRESI-MS *m/z* 595.3015 ($[M+H]^+$, requires 595.3009); UV (H_2O) λ_{max} 266 nm.

2’,3’-*O*-Isopropylidene-*N*[1]-[5’’-*O*-(bis(2-cyanoethyl)phosphate)pentyl] inosine (9)

Compound **5** (70 mg, 0.18 mmol) was coevaporated with anhydrous benzene (3 × 2 mL) and then dissolved in anhydrous THF (1.0 mL). To this solution $(iPr)_2NP(OCE)_2$ (57 mg, 0.21 mmol) and 1*H*-tetrazole (38 mg, 0.54 mmol) were added and the mixture was stirred at room temperature for 2 h. After the completion of the reaction (TLC monitoring: $CHCl_3/CH_3OH$, 9.5:0.5), *t*-BuOOH (5.5 M in decane, 0.33 mL, 1.8 mmol) was added and the reaction was stirred for 2 h at room temperature (TLC monitoring: $AcOEt/CH_3OH$, 8.5:1.5). The solvents were evaporated under reduced pressure and the crude product was purified over a silica gel column eluted with increasing

amounts of CH_3OH in AcOEt (from 0 to 5%). The fractions were collected and the solvents removed under reduced pressure, affording the pure compound **9** (71 mg, 68% over two steps).

Oil; ^{1}H NMR (400 MHz, CD_3OD, assignments by HH-COSY experiment) δ 8.32 (s, 1H, 2-H), 8.31 (s, 1H, 8-H), 6.16 (d, J = 2.9 Hz, 1H, 1'-H), 5.29-5.23 (m, 1H, 2'-H), 5.03-4.98 (m, 1H, 3'-H), 4.38-4.32 (m, 1H, 4'-H), 4.30-4.23 (m, 4H, 2 x $CNCH_2CH_2OP$), 4.21-4-15 (m, 2H, CH_2OP), 4.12 (t, J = 7.2 Hz, 2H, CH_2N), 3.76 (dd, J = 12.0, 3.8 Hz, 1H, 5'-H_a), 3.70 (dd, J = 12.0, 4.4 Hz, 1H, 5'-H_b), 2.90 (t, J = 5.8 Hz, 2H, CH_2CN), 1.90-1.74 (complex signal, 4H, 2 x CH_2), 1.59 (s, 3H, CH_3), 1.55-1-45 (m, 2H, CH_2), 1.37 (s, 3H, CH_3). ^{13}C NMR (100 MHz, CD_3OD) δ 158.3, 149.7, 148.6, 141.3, 125.3, 118.5, 115.3, 92.4, 88.5, 85.9, 82.9, 69.9, 64.3, 63.3, 47.7, 30.6, 30.5, 30.0, 27.5, 25.3, 23.5, 20.2, 20.1.^{31}P NMR (202 MHz, CD_3OD) δ -1.60 (s). HRESI-MS m/z 581.2120 ($[M+H]^+$, requires 581.2125); UV (H_2O) λ_{max} 267 nm.

2',3'-*O*-Isopropylidene-*N*^{1}-[5''-*O*-((2-cyanoethyl)phosphate)pentyl] inosine (10)

Compound **9** (20 mg, 0.034 mmol) was dissolved in a mixture of TEA/pyridine (1:1, 1.0 mL) and the solution was kept at room temperature for 16 h (TLC monitoring: AcOEt/MeOH, 7:3). The solvents were evaporated under reduced pressure ant the crude was coevaporated with pyridine (3 × 1 mL) to obtain compound **10** (18 mg, 90%) as pyridinium salt that was used for the next synthetic step without purification.

Oil. ^{1}H NMR (400 MHz, D_2O, assignments by HH-COSY experiment) δ 8.56-8.50 (m, 2H, Py), 8.34 (s, 1H, 2-H), 8.30 (s, 1H, 8-H), 7.90-7.80 (m, 1H, Py), 7.48-7.40 (m, 2H, Py), 6.17 (d, J = 2.8 Hz, 1H, 1'-H), 5.30-5.25 (m, 1H, 2'-H), 5.03-5.98 (m, 1H, 3'-H), 4.37-4.31 (m, 1H, 4'-H), 4.12 (t, J = 7.2 Hz, 2H, CH_2N), 4.03-3.96 (m, 2H, CH_2OP), 4.02-3.96 (m, 2H, $CNCH_2CH_2O$), 3.75 (dd, J = 11.9, 3.9 Hz, 1H, 5'-H_a), 3.69 (dd, J = 11.9, 4.5 Hz, 1H, 5'-H_b), 2.77 (t, J = 6.0 Hz, 2H, CH_2CN), 1.88-1.78 (m, 2H, CH_2), 1.74-1.65 (m, 2H, CH_2), 1.59 (s, 3H, CH_3), 1.54-1.44 (s, 3H, CH_2), 1.37 (s, 3H, CH_3). ^{13}C NMR (100 MHz, CD_3OD) δ 158.2, 149.9, 149.5, 148.8, 141.2, 135.6, 124.9, 123.9, 118.6, 115.0, 92.7, 88.4, 86.2, 83.5, 69.5, 64.1, 63.3, 47.5, 30.9, 30.4, 30.2, 27.3, 25.4, 23.5, 20.1, 19.8. ^{31}P NMR (202 MHz, CD_3OD) δ -1.68 (s). HRESI-MS m/z 526.1709 ($[M-H]^-$, requires 526.1703); UV (H_2O) λ_{max} 266 nm.

2',3'-*O*-Isopropylidene-*N*^{1}-(5''-*O*-phosphatepentyl)inosine (11)

Compound **9** (50 mg, 0.086 mmol) was dissolved in methanol/concentrated aqueous ammonia (1:1, 1.0 mL) and the reaction was allowed to stir at 50 °C for 16 h (TLC monitoring: isopropanol/ammonia/water, 6:3:1). The solvents were removed under reduced pressure and the crude was purified by a C-18 reversed-phase silica gel column, with increasing amount of CH_3OH

in water (from 0 to 50%). The fractions were collected and the solvents removed under reduced pressure, affording pure compound **11** (30 mg, 72%) as ammonium salt.

Amorphous white solid. ^{1}H NMR (400 MHz, D_2O, assignments by HH-COSY experiment) δ 8.41 (s, 1H, 2-H), 8.29 (s, 1H, 8-H), 6.29 (bs, 1H, 1'-H), 5.51-5.46 (m, 1H, 2'-H), 5.17-5-12 (m, 1H, 3'-H), 4.55-4.49 (m, 1H, 4'-H), 4.17 (t, J = 7.0 Hz, 2H, CH_2N), 3.92-3.85 (m, 2H, CH_2OP), 3.84-3.74 (m, 2H, 5'-$H_{a,b}$), 1.90-1.80 (m, 2H, CH_2), 1.73-1-66 (complex signal, 5H, CH_2 and CH_3), 1.51-1-43 (complex signal, 5H, CH_2 and CH_3). ^{13}C NMR (100 MHz, D_2O) δ 157.9, 148.7, 147.3, 140.3, 123.7, 114.9, 90.3, 86.5, 83.6, 80.9, 80.7, 65.3, 61.4, 47.5, 29.3, 29.2, 28.4, 25.9, 24.2, 22.0. ^{31}P NMR (202 MHz, CD_3OD) δ 1.84 (s). HRESI-MS m/z 473.1441 ([M-H]$^-$, requires 473.1437); UV (H_2O) λ_{max} 265 nm.

5'-*O*-*tert*-Butyldimethylsilyl-2',3'-*O*-isopropylidene-1-(2,4-dinitrophenyl)inosine(13)

To a solution of **12** (1.2 g, 2.8 mmol) in DMF (12 mL), K_2CO_3 (1.56 g, 1.1 mmol) and 2,4-dinitrochlorobenzene (2.3 g, 1.1 mmol) were added and the reaction was stirred for 4 h at 80 °C (TLC monitoring $CHCl_3$/MeOH, 9.5:0.5). The solvent was evaporated under reduced pressure and the crude was purified over a silica gel column eluted with increasing amounts of CH_3OH in $CHCl_3$ (from 0 to 5%). The fractions were collected and the solvents removed under reduced pressure, affording pure compound **13** (1.0 g, 63%).

Yellow solid; ^{1}H NMR (400 MHz, $CDCl_3$, assignments by HH-COSY experiment) δ 8.97 (s, 1H, ArH), 8.64-8.62 (m, 1H, ArH), 8.12-8.11 (m, 1H, 2-H), 8.06-8.05 (m, 1H, 8-H), 7.77-7.68 (m, 1H, ArH), 6.17-6.15 (m, 1H, 1'-H), 5.11-5.03 (m,1H, 2'-H), 4.93-4.90 (m, 1H, 3'-H), 4.49-4.45 (m, 1H, 4'-H), 3.92-3.79 (m, 2H, 5'-H), 1.63 (s, 3H, isopropylidene), 1.39 (s, 3H, isopropylidene), 0.86-0.83 (s, 9H, $(CH_3)_3C$), 0.043 (s, 3H, CH_3), 0.027 (s, 3H, CH_3) ppm. ^{13}C NMR (100 MHz, $CDCl_3$) δ 155.1, 148.1, 147.0, 145.1, 139.0, 135.6, 132.9, 128.9, 124.3, 121.3, 114.3, 114.1, 92.0, 91.4, 87.5, 87.0, 85.7, 81.3, 63.6, 27.2, 25.8, 25.3, 18.3, -5.4; HRESI-MS m/z 589.2085 ([M+H]$^+$, requires 589.2078); UV ($CHCl_3$) λ_{max} 264 nm.

5'-*O*-*tert*-Butyldimethylsilyl-2',3'-*O*-isopropylidene-*N*^{1}-(5-hydroxypentyl)inosine (14)

To a solution of **13** (0.50 g, 0.85 mmol) in DMF (2.0 mL) 5-amino-1-pentanol (0.90 g, 8.5mmol) was added and the mixture was stirred at 50 °C for 16 h (TLC monitoring $CHCl_3$/MeOH, 9.5:0.5). The solvent was evaporated under reduced pressure and the crude was purified over a silica gel column eluted with increasing amounts of CH_3OH in $CHCl_3$ (from 0 to 5%). The fractions were

collected and the solvents removed under reduced pressure, affording pure compound **14** (0.33 g, 77%).

Yellow oil; ^{1}H NMR (400 MHz, CD_3OD, assignments by HH-COSY experiment) δ 8.30 (s, 1H, 2-H), 8.20 (s, 1H, 8-H), 6.15 (d, J = 2.0 Hz, 1H, 1'-H), 5.31-5.29 (m, 1H, 2'-H), 4.96-4.93 (m, 1H, 3'-H), 4.35-4.33 (m, 1H, 4'-H), 4.13-4.06 (m, 2H, CH_2N), 3.84 (dd, J = 11.2, 4.1 Hz, 2H, 5'-H_a), 3.77 (dd, J = 11.2, 4.7 Hz, 2H, 5'-H_b), 3.54 (t, J = 6.0 Hz, 2H, CH_2O), 1.81-1.75 (m, 2H, CH_2), 1.60-1.53 (complex signal, 3H, CH_2 and isopropylidene), 1.46-1.38 (m, 2H, CH_2), 1.36 (s, 3H, isopropylidene), 0.81 (s, 9H, $(CH_3)_3C$), -0.0010 (s, 3H, CH_3), -0.0070 (s, 3H, CH_3). ^{13}C NMR (100 MHz, CD_3OD) δ 158.1, 149.5, 148.5, 140.8, 125.3, 115.0, 92.5, 88.9, 86.1, 82.8, 64.7, 62.5, 47.8, 33.0, 30.5, 27.4, 26.3, 25.5, 23.8, 19.1, -5.3 ppm; HRESI-MS m/z 509.2789 ($[M+H]^+$, requires 509.2795); UV (H_2O) λ_{max} 265 nm.

5'-*O*-*tert*-Butyldimethylsilyl-2',3'-*O*-isopropylidene-*N*^{1}-(5-acetoxypentyl)inosine (15)

Compound **14** (0.33 g, 0.65 mmol) was dissolved in a mixture of Ac_2O-pyridine (1:1, 2.0 mL) and the solution was kept at room temperature for 2 h (TLC monitoring: $CHCl_3$/MeOH, 9.5:0.5).The solvents were evaporated under reduced pressure to give compound **15** (0.35 g, 99%) that was used for the next synthetic step without purification.

Yellow oil; ^{1}H NMR (400 MHz, CD_3OD, assignments by HH-COSY experiment) δ= 8.31 (s, 1H, 2-H), 8.21 (s, 1H, 8-H), 6.16 (d, J = 2.0 Hz, 1H, 1'-H), 5.32-5.30 (m, 1H, 2'-H), 4.97-4.95 (m, 1H, 3'-H), 4.37-4.35 (m, 1H, 4'-H), 4.15-4.09 (m, 2H, CH_2N), 4.07-4.04 (t, J = 6.3 Hz, 2H, CH_2O), 3.85 (dd, J = 11.3, 4.1 Hz, 1H, 5'-H_a), 3.78 (dd, J = 11.3, 4.7 Hz, 1H, 5'-H_b), 2.00 (s, 3H, CH_3CO), 1.86-1.76 (m, 2H, CH_2), 1.73-1.65 (m, 2H, CH_2), 1.58 (s, 3H, isopropylidene), 1.48-1.40 (m, 2H, CH_2), 1.37 (s, 3H, isopropylidene), 0.82 ($(CH_3)_3C$), 0.0050 (s, 3H, CH_3), -0.0020 (s, 3H, CH_3). ^{13}C NMR (100 MHz, CD_3OD)δ 172.5, 157.9, 149.7, 149.4, 148.3, 140.75, 125.2, 114.9, 92.3, 88.8, 86.0, 82.7, 65.1, 64.6, 47.5, 30.2, 29.1, 27.5, 26.3, 25.5, 23.8, 22.0, 20.8, 20.8, 19.0, -5.2; HRESI-MS m/z 551.2909 ($[M+H]^+$, requires 551.2901); UV (H_2O) λ_{max} 266 nm.

2',3'-*O*-Isopropylidene-*N*^{1}-(5-acetoxypentyl)inosine (16)

Compound **15** (0.30 g, 0.54 mmol) was dissolved in MeOH (10 mL) and then NH_4F (0.40 g, 11 mmol) was added. The reaction was refluxed for 16 h (TLC monitoring: $CHCl_3$/MeOH, 9.5:0.5). The solvent was evaporated under reduced pressure and the crude was purified over a silica gel

column eluted with increasing amounts of CH_3OH in $CHCl_3$ (from 0 to 10%). The fractions were collected and the solvents removed under reduced pressure, affording pure compound **16** (0.21 g, 91%).

Yellow oil; ^1H NMR (400 MHz, CD_3OD, assignments by HH-COSY experiment) δ 8.31 (s, 1H, 2-H), 8.30 (s, 1H, 8-H), 6.13 (d, J = 2.4 Hz, 1H, 1'-H), 5.24-5.22 (m, 1H, 2'-H), 4.99-4.97 (m, 1H, 3'-H), 4.32-4.30 (m, 1H, 4'-H), 4.08 (t, J = 7.4 Hz, 2H, CH_2N), 4.03 (*t*, J = 6.3 Hz, 2H, CH_2O), 3.76-3.68 (m, 2H, 5'-$H_{a,b}$), 1.98 (s, 3H, CH_3CO), 1.79-1.76 (m, 2H, CH_2), 1.66-1.61 (m, 2H, CH_2), 1.56 (s, 3H, isopropylidene), 1.44-1.38 (m, 2H, CH_2), 1.34 (s, 3H, isopropylidene). ^{13}C NMR (100 MHz, CD_3OD) δ 172.7, 158.0, 149.6, 148.4, 141.1, 125.2, 115.1, 92.2, 88.3, 85.8, 82.7, 65.2, 63.2, 47.6, 30.2, 29.1, 27.5, 25.5, 23.8, 20.8; HRESI-MS *m/z* 437.2041 ($[M+H]^+$, requires 437.2036); UV (H_2O) λ_{max} 267 nm.

2',3'-*O*-Isopropylidene-N^1-(5-acetoxypentyl)inosine 5'-bis(2-cyanoethyl) phosphate (17)

Compound **16** (0.20 g, 0.46 mmol) was coevaporated with anhydrous benzene (3 x 2 mL) and then dissolved in anhydrous THF (4.0 mL). To this solution $(iPr)_2NP(OCE)_2$ (0.25 g, 0.92 mmol) and tetrazole (0.10 g, 1.4 mmol) were added and the mixture was stirred at room temperature for 2 h. After the completion of the reaction (TLC monitoring: $CHCl_3/CH_3OH$, 9.5:0.5), *t*-BuOOH (5.5 M in decane, 0.80 mL, 4.6 mmol) was added and the reaction was stirred for 2 h at room temperature (TLC monitoring: $CHCl_3/CH_3OH$, 9.5:0.5). The solvents were evaporated under reduced pressure and the crude was purified over a silica gel column eluted with increasing amounts of CH_3OH in $CHCl_3$ (from 0 to 10%). The fractions were collected and the solvents removed under reduced pressure, affording the pure compound **17** (0.23 g, 80% over two steps).

Yellow oil; ^1H NMR (400 MHz, CD_3OD, assignments by HH-COSY experiment) δ 8.32 (s, 1H, 2-H), 8.20 (s, 1H, 8-H), 6.23 (d, J = 1.9 Hz, 1H, 1'-H), 5.46-5.41 (m, 1H, 2'-H), 5.16-5.10 (m, 1H, 3'-H), 4.52-4.46 (m, 1H, 4'-H), 4.40-4-05 (complex signal, 10H, 2 x CH_2OP, 5'-$H_{a,b}$, CH_2O, CH_2N), 2.93 (t, J = 5.8 Hz, 4H, 2 x CH_2CN), 2.00 (s, 3H, CH_3), 1.86-1.77 (m, 2H, CH_2), 1.74-1.64 (m, 2H, CH_2), 1.60 (s, 3H, isopropylidene), 1.50-1.41 (m, 2H, CH_2), 1.39 (s, 3H, isopropylidene). ^{13}C NMR (100 MHz, CD_3OD) δ 172.5, 158.1, 149.4, 148.1, 141.5, 125.6, 118.5, 115.5, 92.4, 88.6, 85.7, 82.5, 66.8, 64.9, 62.6, 47.7, 30.3, 29.5, 27.7, 25.4, 22.7, 20.9; ^{31}P NMR (202 MHz, CD_3OD) δ -1.53 (s). HRESI-MS *m/z* 623.2237 ($[M+H]^+$, requires 623.2231); UV (H_2O) λ_{max} 265 nm.

2',3'-*O*-Isopropylidene-N^1-(5-hydroxypentyl)inosine 5'-monophosphate (18)

Compound **17** (0.10 g, 0.16 mmol) was dissolved in methanol/concentrated aqueous ammonia (1:1, 2.0 mL) and the reaction was allowed to stir at 50 °C for 16 h (TLC monitoring: isopropanol/ammonia/water, 6:3:1). The solvents were removed under reduced pressure and the crude was purified by a C-18 reversed-phase silica gel column with increasing amount of CH_3OH in water (from 0 to 50%). The fractions were collected and the solvents removed under reduced pressure, affording the pure compound **18** (55 mg, 70%) as ammonium salt.

Amorphous white solid; ^{1}H NMR (400 MHz, CD_3OD, assignments by HH-COSY experiment) δ 8.49 (s, 1H, 2-H), 8.31 (s, 1H, 8-H), 6.17 (d, J = 3.1 Hz, 1H, 1'-H), 5.35-5.30 (m, 1H, 2'-H), 5.18-5.12 (m, 1H, 3'-H), 4.51-4.47 (m, 1H, 4'-H), 4.14-4.07 (t, J = 7.2 Hz, 2H, CH_2N), 4.04-3.98 (m, 2H, 5'-$H_{a,b}$), 3.55 (t, J = 6.4 Hz, 2H, CH_2O), 1.86-1.76 (m, 2H, CH_2), 1.62-1.54 (complex signal, 5H, CH_2 and isopropylidene), 1.37 (s, 3H, isopropylidene); ^{13}C NMR (100 MHz, CD_3OD), 157.9, 148.7, 147.3, 140.3, 123.7, 114.9, 90.3, 86.5, 83.6, 80.9, 80.7, 65.3, 61.4, 47.5, 29.3, 29.2, 28.4, 25.9, 24.2, 22.0; ^{31}P NMR (202 MHz, CD_3OD) δ 1.84 (s). HRESI-MS m/z 473.1443 ([M-H]$^-$, requires 473.1437); UV (H_2O) λ_{max} 267 nm.

2',3'-*O*-isopropylidene-N^1-pentylinosine cyclic 5',5''-phosphate (19)

Compound **18** (20 mg, 0.040 mmol) was dissolved in DMF (20 mL) and then EDC (9.6 mg, 0.050 mmol) was added. The reaction was allowed to stir for 48 h at room temperature (TLC monitoring: isopropanol/ammonia/water, 6:3:1). After removing the solvent under reduced pressure, the crude was dissolved in 1 mL of TEAB 0.1 M and then purified by HPLC (see General). The fractions containing the title compound were collected, concentrated and finally lyophilized to afford compound **19** (6.6 mg, 30%) as triethylammonium salt.

Amorphous white solid; ^{1}H NMR (400 MHz, D_2O, assignments by HH-COSY experiment) δ 8.43 (s, 1H, 2-H), 8.31 (s, 1H, 8-H), 6.32 (bs, 1H, 1'-H), 5.77-5.72 (m, 1H, 2'-H), 5.21-5-16 (m, 1H, 3'-H), 4.53-4.49 (m, 1H, 4'-H), 4.19 (t, J = 7.3 Hz, 2H, CH_2N), 4.04-3.98 (m, 2H, 5'-$H_{a,b}$), 3.92-3.85 (m, 2H, CH_2OP), 3.22 (q, J = 7.3 Hz, 6H, CH_2 of triethylammonium), 1.90-1.80 (m, 2H, CH_2), 1.75-1.68 (complex signal, 5H, CH_2 and CH_3), 1.53-1-45 (complex signal, 5H, CH_2 and CH_3), 1.29 (t, J = 7.3 Hz, 9H, CH_3 of triethylammonium). ^{13}C NMR (100 MHz, D_2O) δ 157.7, 148.5, 147.6, 140.2, 122.9, 115.2, 90.1, 86.7, 83.4, 80.7, 65.3, 64.7, 47.2, 29.1, 28.6, 28.4, 25.7, 24.1, 22.0; ^{31}P NMR (202 MHz, CD_3OD) δ -1.84 (s). HRESI-MS m/z 455.1341 ([M-H]$^-$, requires 455.1337); UV (H_2O) λ_{max} 269 nm.

N^1-Pentylinosine cyclic 5',5''-phosphate (4)

Compound **19** (5.0 mg, 0.0090 mmol) was dissolved in a mixture of TFA-H_2O (2:8, 0.5 mL) and the reaction was allowed to stir for 16 h at room temperature (TLC monitoring: isopropanol/ammonia/water, 6:3:1). After removing the solvents under reduced pressure, the crude was dissolved in 0.5 mL of TEAB 0.1 M and then purified by HPLC (see General). The fractions containing the title compound were collected, concentrated and finally lyophilized to afford compound **4** (3.7 mg, 80%) as triethylammonium salt.

Amorphous white solid. ^{1}H NMR (400 MHz, D_2O, assignments by HH-COSY experiment) δ 8.39 (s, 1H, 2-H), 8.35 (s, 1H, 8-H), 6.21 (bs, 1H, 1'-H), 5.40-5.35 (m, 1H, 2'-H), 4.53-4.49 (m, 1H, 3'-H), 4.45-4.40 (m, 1H, 4'-H), 4.17 (t, J = 7.1 Hz, 2H, CH_2N), 4.02-3.97 (m, 2H, 5'-$H_{a,b}$), 3.90-3.85 (m, 2H, CH_2OP), 3.25 (q, J = 7.3 Hz, 6H, CH_2 of triethylammonium), 1.88-1.78 (m, 2H, CH_2), 1.67-1.56 (m, 2H, CH_2), 1.46-1.38 (m, 2H, CH_2), 1.31 (t, J = 7.3 Hz, 9H, CH_3 of triethylammonium). ^{13}C NMR (100 MHz, D_2O) δ 156.9, 149.9, 148.2, 140.2, 122.7, 88.5, 83.9, 74.6, 69.9, 65.0, 64.5, 47.4, 30.7, 28.3, 22.0; ^{31}P NMR (202 MHz, CD_3OD) δ -1.88 (s). HRESI-MS m/z 415.1029 ($[M-H]^-$, requires 415.1024); UV (H_2O) λ_{max} 269 nm.
